# The artificial sweetener acesulfame potassium affects the gut microbiome and body weight gain in CD-1 mice

**DOI:** 10.1371/journal.pone.0178426

**Published:** 2017-06-08

**Authors:** Xiaoming Bian, Liang Chi, Bei Gao, Pengcheng Tu, Hongyu Ru, Kun Lu

**Affiliations:** 1 Department of Environmental Health Science, University of Georgia, Athens, Georgia, United States of America; 2 Department of Environmental Sciences and Engineering, University of North Carolina at Chapel Hill, Chapel Hill, North Carolina, United States of America; 3 Department of Population Health and Pathobiology, North Carolina State University, Raleigh, North Carolina, United States of America; Western University of Health Sciences, UNITED STATES

## Abstract

Artificial sweeteners have been widely used in the modern diet, and their observed effects on human health have been inconsistent, with both beneficial and adverse outcomes reported. Obesity and type 2 diabetes have dramatically increased in the U.S. and other countries over the last two decades. Numerous studies have indicated an important role of the gut microbiome in body weight control and glucose metabolism and regulation. Interestingly, the artificial sweetener saccharin could alter gut microbiota and induce glucose intolerance, raising questions about the contribution of artificial sweeteners to the global epidemic of obesity and diabetes. Acesulfame-potassium (Ace-K), a FDA-approved artificial sweetener, is commonly used, but its toxicity data reported to date are considered inadequate. In particular, the functional impact of Ace-K on the gut microbiome is largely unknown. In this study, we explored the effects of Ace-K on the gut microbiome and the changes in fecal metabolic profiles using 16S rRNA sequencing and gas chromatography-mass spectrometry (GC-MS) metabolomics. We found that Ace-K consumption perturbed the gut microbiome of CD-1 mice after a 4-week treatment. The observed body weight gain, shifts in the gut bacterial community composition, enrichment of functional bacterial genes related to energy metabolism, and fecal metabolomic changes were highly gender-specific, with differential effects observed for males and females. In particular, ace-K increased body weight gain of male but not female mice. Collectively, our results may provide a novel understanding of the interaction between artificial sweeteners and the gut microbiome, as well as the potential role of this interaction in the development of obesity and the associated chronic inflammation.

## Introduction

As widely used food additives and sugar substitutes, artificial sweeteners can enhance flavor and simultaneously reduce calorie intake. Some epidemiological studies have shown that artificial sweeteners are beneficial for weight loss and to those who suffer from glucose intolerance and type 2 diabetes mellitus [1]. However, accumulating evidence in recent years suggests that artificial sweetener consumption could perturb human metabolism, especially glucose regulation [2, 3]. Artificial sweeteners have been found to cause glucose intolerance and induce metabolic syndrome and are also associated with higher body weight gain [3–6]. These findings suggest that artificial sweeteners may increase the risk of obesity. However, the specific mechanism through which artificial sweeteners dysregulate the host metabolism remains elusive.

Recently, much attention has been paid to the regulating effects of the gut microbiota on the host health. The gut microbiome is deeply involved in host metabolism and plays a crucial role in food digestion and energy homeostasis in the human body [7–9]. Moreover, commensal microflora colonization is necessary for immune system development, enteric nerve regulation and pathogen prevention [10–13]. However, multiple environmental factors, such as diet, antibiotics and heavy metals, can disrupt the ecological balance in the gut [14–16]. The dysbiosis of the gut microbiome is associated with a series of human diseases, including obesity, diabetes, and inflammatory bowel disease [9, 17]. A previous study found that consumption of Splenda, a nonnutritive sweetener containing 1% sucralose, impaired the growth of gut bacteria in rats [18]. In addition, a recent study found that non-caloric artificial sweeteners, such as saccharin, impaired glucose tolerance by modulating the composition of gut bacteria [3]. However, the specific effects of artificial sweeteners on the gut microbiota and their metabolism are still largely unknown. In addition, chronic inflammation commonly occurs in obesity and diabetes, and gut bacteria can produce numerous pro-inflammatory mediators, raising the question of the potential role of artificial sweetener-disrupted gut bacteria in eliciting host inflammation.

Acesulfame-K (Ace-K) is one of the major low-calorie artificial sweeteners in the modern diet. Although its toxicity data reported to date are considered inadequate [19], previous studies have found that Ace-K is genotoxic and can inhibit glucose fermentation by intestinal bacteria [20, 21]. Also, Ace-K, like sodium saccharin and sodium cyclamate, belongs to sulfonamides, a chemical class associated with antimicrobial activity[22]. A recent study found that overall bacterial diversity was different across nonconsumers and consumers of artificial sweeteners, including Ace-K and aspartame, in heathy human adults in the United States [23], but the consumption and doses of the artificial sweeteners were only estimated based on a four-day food record. How Ace-K perturbs the gut microbiome and whether it leads to functional changes in the gut microbiota is still largely unknown. In particular, the interaction between the host and gut microbiome is complicated, and many host characteristics can influence the responses of the gut microbiome to external stimuli. Among these factors, gender emerges as important yet unexplored influence. Previous research has demonstrated that females and males have dramatically different physiological conditions and gut microbiome patterns [24, 25]. We have demonstrated gender-dependent responses of the gut microbiome to xenobiotics, such as arsenic and organophosphates [24, 26]. For example, changes in gut bacteria and associated metabolic functions were different between male and female mice exposed to arsenic, which is consistent with gender-specific disease outcomes [27–29]. Therefore, the examination of gender-specific gut microbiome responses to Ace-K consumption would be highly informative.

In this study, we investigated the effects of Ace-K on the gut microbiome and the changes in the fecal metabolome using 16S rRNA sequencing and gas chromatography-mass spectrometry (GC-MS) metabolomics. We found that Ace-K consumption perturbed the gut microbiome of CD-1 mice after a 4-week treatment. The observed body weight gain, shifts in the gut bacterial community composition, enrichment of bacterial functional genes and fecal metabolomic changes were highly gender dependent. Specifically, Ace-K increased the body weight gain in male but not female mice. The functional genes involved in energy metabolism were activated in male mice but inhibited in female mice. Moreover, differential changes in fecal metabolic profiles were observed between male and female animals. Taken together, these results may provide novel insights into understanding the functional interaction between artificial sweeteners and the gut microbiome and the role of this interaction in the development of obesity and chronic inflammation.

## Materials and methods

### Animals and exposure

CD-1 mice (~7 weeks old) were purchased from Charles River and provided a standard pelleted rodent diet and tap water ad libitum under the following environmental conditions: 22°C, 40–70% humidity, and a 12:12 hour light:dark cycle. All 20 mice (10 male and 10 female) were housed in the University of Georgia animal facility for 1 week before the experiments. Then, the mice were randomly assigned to the control and Ace-K groups (five male and five female mice in each group). Water (control) and artificial sweeteners were administered to the mice (~8 weeks old) through gavage for 4 weeks, with the dose of 37.5 mg/kg body weight/day. This dose was equivalent to or much lower than those used in previous animal studies [20, 30]. Body weight was measured before and after the treatment. There was no any statistical difference in the initial body weight between the control and Ace-K group for either male or female mice (males: 26.8±1.1 g and 26.4±1.1 g for the control and Ace-K group; females: 22.8±1.6 g and 22.4±1.1 g for the control and Ace-K group). Mice were euthanized with CO_2_ in an appropriate chamber by trained personnel. All experiments were approved by the University of Georgia Institutional Animal Care and Use Committee. The animals were treated humanely and with regard for alleviation of suffering.

### 16S rRNA gene sequencing

DNA was isolated from frozen fecal pellets collected at different time points using a PowerSoil DNA Isolation Kit (Mo Bio Laboratories) according to the manufacturer’s instruction, and the resultant DNA was quantified and stored at -80°C for further analysis. Purified DNA (1 ng) was used to amplify the V4 region of the 16S rRNA of bacteria using the universal primers 515 (5’-GTGCCAGCMGCCGCGGTAA) and 806 (5’-GGACTACHVGGGTWTCTAAT). Individual samples were barcoded, pooled to construct a sequencing library, and then sequenced by Illumina MiSeq at the Georgia Genomics Facility to generate pair-end 250 × 250 (PE250, v2 kit) reads to a depth of at least 25,000 reads per sample. The raw mate-paired FASTQ files were merged and quality-filtered using Geneious 8.0.5 (Biomatters, Auckland, New Zealand) with the error probability limit set at 0.01. The data were then analyzed using quantitative insights into microbial ecology (QIIME, version 1.9.1). UCLUST was used to obtain the operational taxonomic units (OTUs) with 97% sequence similarity. The data were assigned at five different levels: phylum, class, order, family and genus.

### Functional gene enrichment analysis

The Phylogenetic Investigation of Communities by Reconstruction of Unobserved States (PICRUSt) (Galaxy Version 1.0.0) was first used to analyze the enrichment of functional genes in the gut microbiome of each group [31]. PICRUSt can accurately profile the functional genes of bacterial communities based on the marker genes from 16S sequencing data and a database of reference genomes [32–34]. PICRUSt has been widely used in microbiome functional gene enrichment analysis, with ~95% accuracy being reported for bacterial metagenomes [32–34]. The results from PICRUSt were then imported into the Statistical Analysis of Metagenomic Profiles (STAMP) (version 2.1.3) package for further statistical analysis and visualization [35].

### Metabolomics analysis

Metabolites were extracted from fecal samples using methanol and chloroform as described previously [16]. Briefly, 20 mg of feces was vortexed with 1 ml of methanol/chloroform/water solution (2:2:1) for 1 hour, followed by centrifugation at 3,200 x g for 15 minutes. The resultant upper and lower phases were transferred to a gas chromatography (GC) vial, dried for approximately 4 hours in a SpeedVac, and derivatized using N,O-Bis(trimethylsilyl)trifluoroacetamide (BSTFA). An Agilent 6890/5973 GC-MS system equipped with a DB-5ms column (Agilent, Santa Clara, CA) was used to conduct the metabolomic profiling and capture all detectable metabolite features over a mass range from 50 to 600 m/z. The XCMS online tool was used to identify and align peaks and calculate the accumulated peak intensity.

### Statistical analysis

The difference in the individual gut microbiota between day 0 and week 4 was assessed using mothur software [36]. A heat map was used to visualize the clustering of enriched functional genes. A two-tailed Welch’s t-test (p<0.05) was used to assess the differences in the metabolic profiles between the control and artificial sweetener groups. Additionally, partial least squares discriminant analysis (PLS-DA) was performed to examine the differences in the metabolomes of the different groups.

## Results

### 1. Ace-K increased the body weight gain of male but not female mice

**Fig 1:**
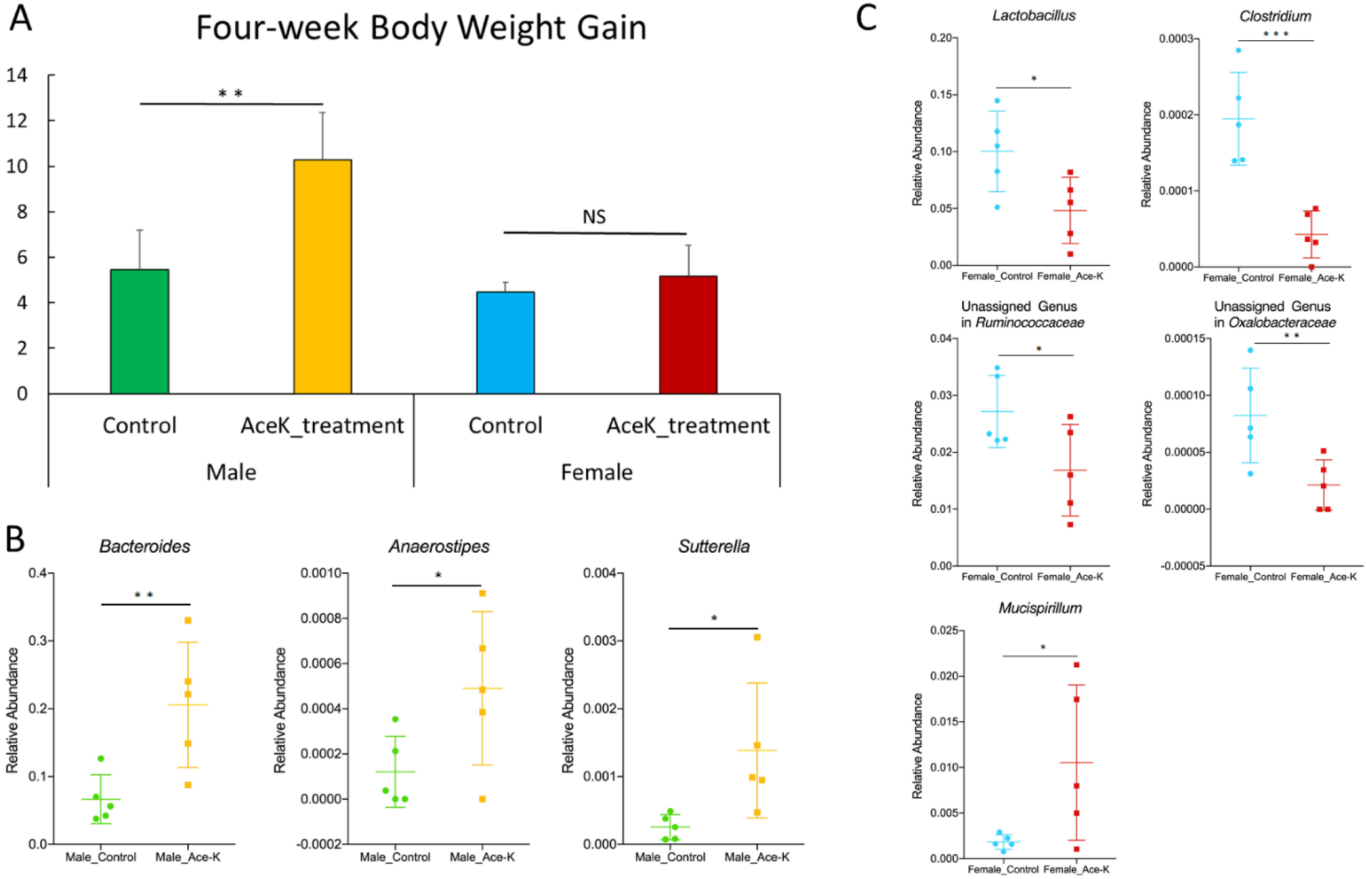
Effects of four weeks of Ace-K consumption on the body weight gain and gut microbiome composition of CD-1 mice. (A) The body weight gain of Ace-K-treated male mice was significantly higher than that of the control male mice, while the body weight gain of female mice was not significantly different from that of the controls. (B) Ace-K consumption altered the composition of gut bacteria in female mice. The abundances of *Lactobacillus*, *Clostridium*, an unassigned *Ruminococcaceae* genus and an unassigned *Oxalobacteraceae* genus were significantly decreased, and the abundance of *Mucispirillum* was increased after Ace-K consumption. (C) Ace-K consumption altered the composition of gut bacteria in male mice. The abundances of *Bacteroides*, *Anaerostipes* and *Sutterella* were significantly increased after Ace-K consumption (*p<0.05, **p<0.01, ***p<0.001, N.S. p>0.05).

We first examined the body weight gain of mice after a four-week treatment. Clearly, the treated male mice exhibited much higher body weight gain than the control male mice (10.28 g versus 5.44 g, p<0.01). For female mie, the body weight gain was not significantly different between the control and treated animals, as shown in Fig 1A.

### 2. Ace-K altered the gut microbiome components in a gender-specific manner

Given that Ace-K induced gender-dependent body weight gain in animals and considering the crucial role of gut bacteria in host energy homeostasis, we further explored whether Ace-K has different effects on the gut microbiota of male and female mice. Fig 1B and 1C show the genera of gut bacteria that were significantly changed (p<0.05) in female and male mice. Notably, *Bacteroides* was highly increased in Ace-K-treated male mice, along with significant changes in two other genera, *Anaerostipes* and *Sutterella*, as shown in Fig 1C. However, in female mice, the four-week Ace-K treatment dramatically decreased the relative abundance of multiple genera, including *Lactobacillus*, *Clostridium*, an unassigned *Ruminococcaceae* genus and an unassigned *Oxalobacteraceae* genus, and increased the abundance of *Mucispirillum*, as shown in Fig 1B. These results indicated that Ace-K perturbed the gut microbiome composition in a gender-dependent manner.

### 3. Ace-K induced gender-specific changes in functional genes related to energy metabolism

**Fig 2:**
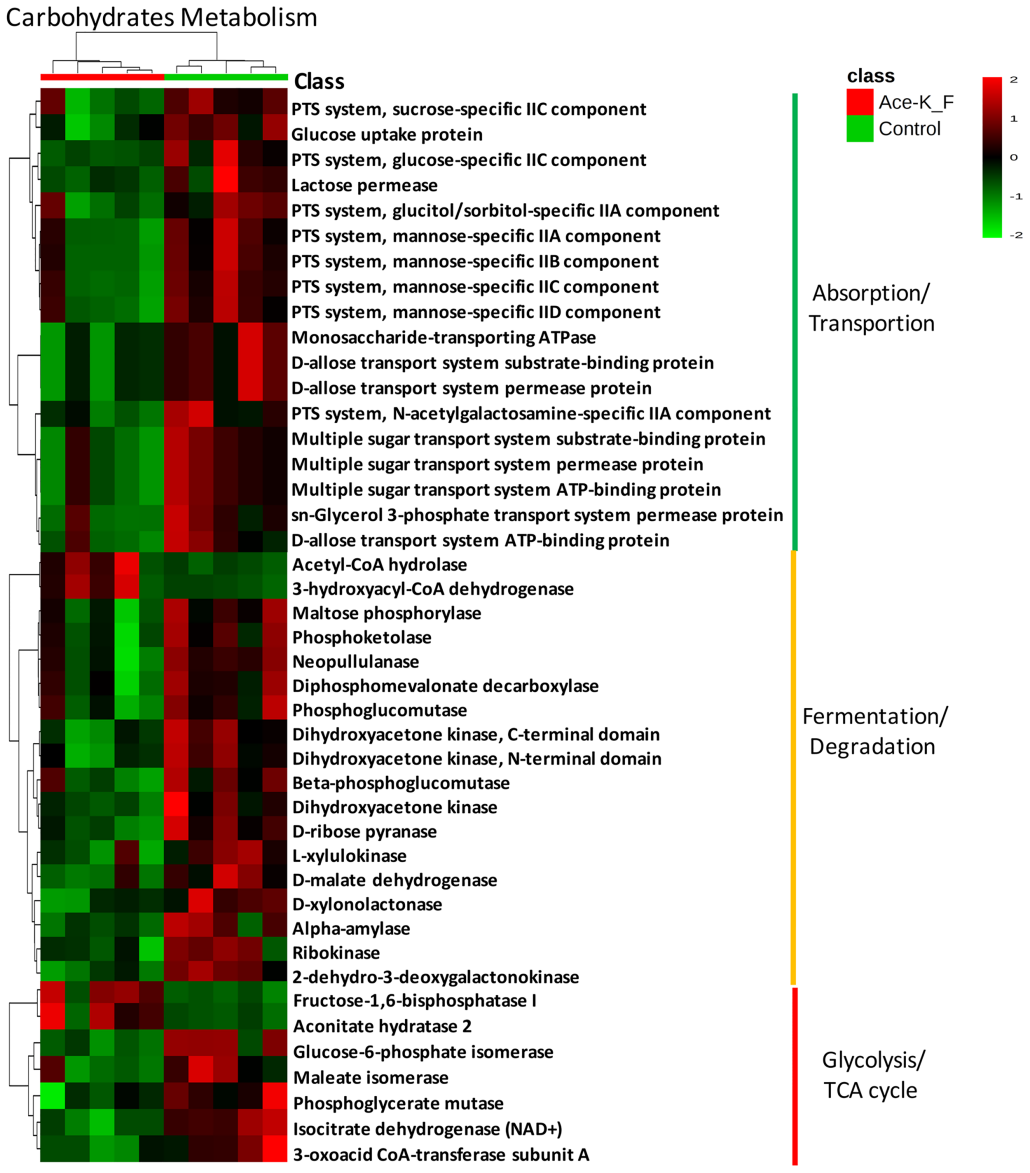
Functional gene enrichment analysis showing that functional genes related to carbohydrate metabolism were significantly decreased in Ace-K-treated female mice (p<0.05 for all genes listed here).

Different gut microbiome composition profiles are generally associated with different functional gene pools. Therefore, we further investigated the functional gene alterations induced by Ace-K consumption. In Ace-K-treated female mice, many of the genes involved in key energy metabolism pathways were decreased (Fig 2), which is consistent with the decrease in multiple genera in the female mice. The relative abundances of numerous genes involved in carbohydrate absorption or transport, including glucose uptake protein, lactose permease, monosaccharide-transporting ATPase, components of multiple sugar and D-allose transport systems, and different types of phosphotransferase systems, were significantly reduced. Likewise, multiple polysaccharide hydrolysis and degradation genes, such as L-xylulokinase, D-xylonolactonase and alpha-amylase, were also decreased in female animals administered Ace-K.

**Fig 3:**
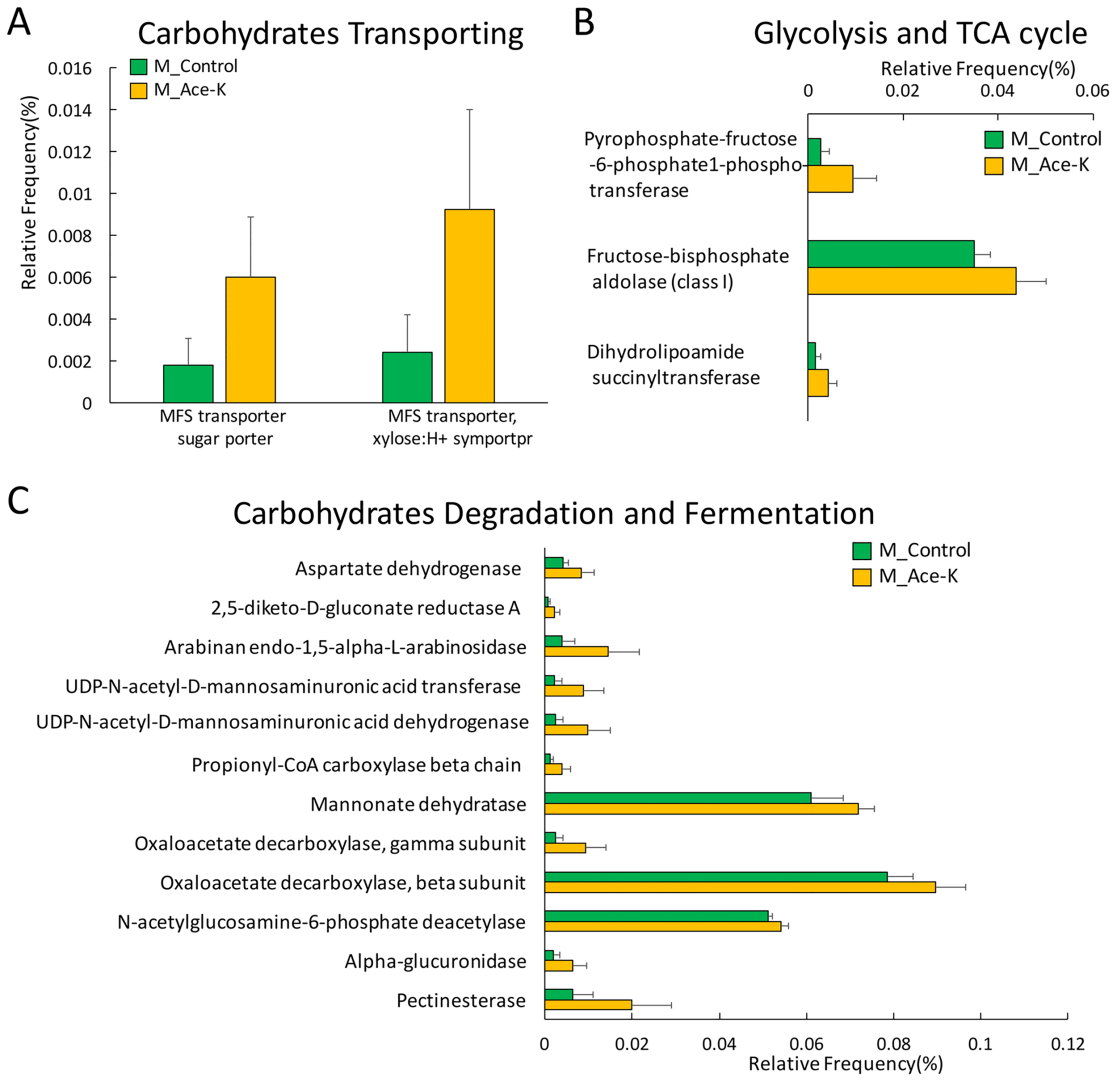
Functional gene enrichment analysis showing that functional genes related to carbohydrate metabolism were significantly increased in Ace-K-treated male mice (p<0.05). Genes involved in carbohydrate transport (A), glycolysis and the TCA cycle (B), as well as carbohydrate degradation and fermentation (C), were consistently increased.

In contrast, carbohydrate absorption and metabolism pathways were activated in treated male animals, which corresponded to the large increase in *Bacteroides*. Specifically, genes involved in sugar and xylose transport, glycolysis and the TCA cycle were increased, as shown in Fig 3A and 3B. In addition, the abundances of multiple genes involved in carbohydrate metabolism and fermentation pathways were also consistently increased (Fig 3C). Taken together, the functional gene enrichment identified by PICRUSt indicates that Ace-K consumption can induce gender-specific responses of carbohydrate metabolism in the gut microbiome.

### 4. Ace-K increased the abundance of genes related to lipopolysaccharide (LPS) synthesis

**Fig 4:**
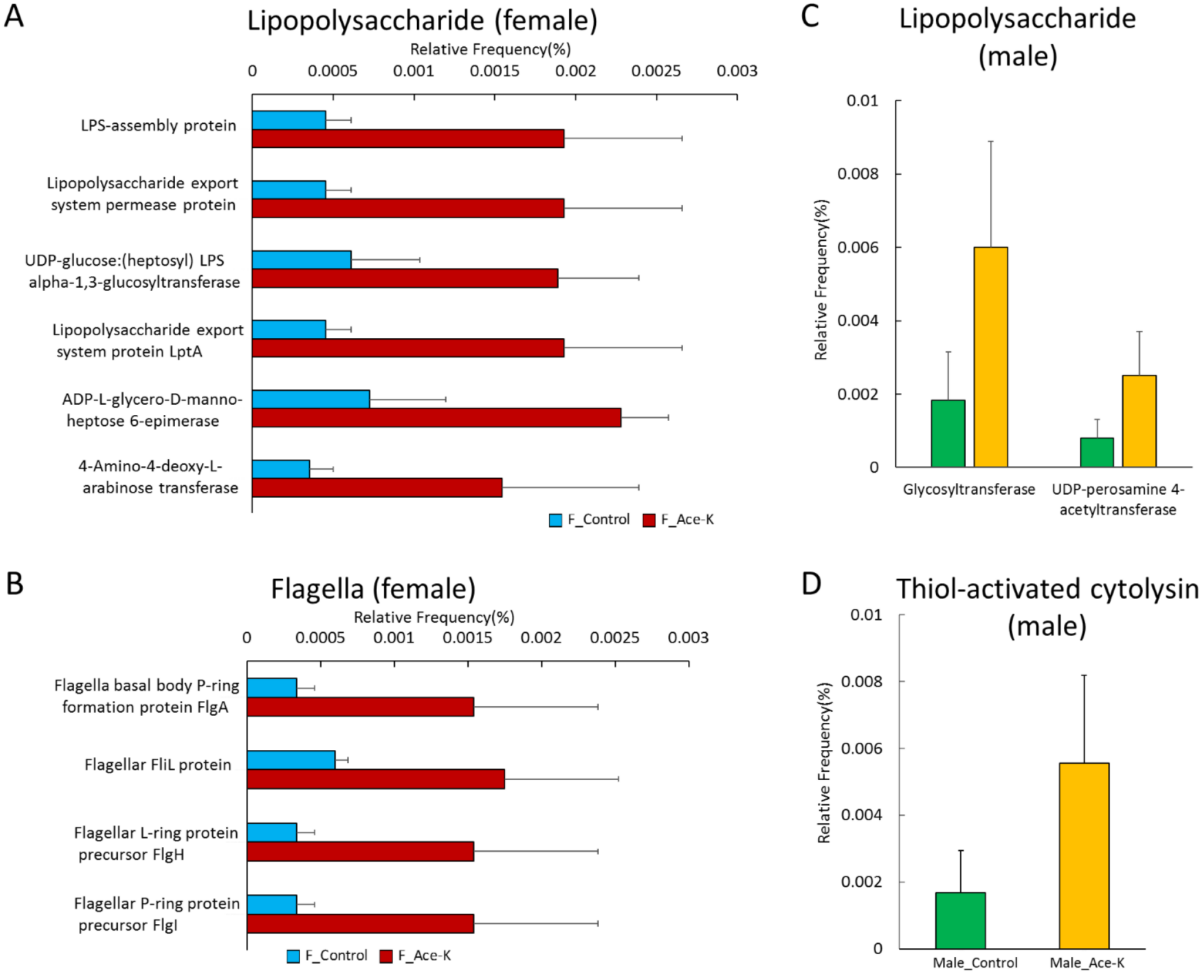
Multiple genes encoding pro-inflammatory mediators were significantly increased in male and female mice after Ace-K consumption (p<0.05). Genes encoding LPS metabolism proteins (A) and flagella components (B) were increased in Ace-K-treated female mice. Genes encoding LPS metabolism proteins (C) and thiol-activated cytolysin (D) were increased in Ace-K-treated male mice.

Since gut microbiome imbalance and metabolic syndrome are generally associated with systemic chronic inflammation, we further investigated whether the perturbation of the composition and functional genes of gut microbiota would contribute to inflammation. As shown in Fig 4A and 4C, many genes involved in LPS synthesis were increased in mice after Ace-K consumption. For example, in Ace-K-treated female mice, LPS synthesis-related genes, including UDP-glucose:(heptosyl) LPS alpha-1,3-glucosyltransferase, ADP-L-glycero-D-manno-heptose 6-epimerase, amino-4-deoxy-L-arabinose transferase, UDP-D-GlcNAcA oxidase and UDP-GlcNAc3NAcA epimerase, were significantly increased. LPS-export genes, LPS export system protein LptA and LPS export system permease protein, and an LPS-assembly protein were also increased by Ace-K treatment. In addition, multiple genes encoding flagella components, including flagella basal body P-ring formation protein FlgA, flagellar L-ring protein precursor FlgH, flagellar P-ring protein precursor FlgI and flagellar FliL protein, were increased, as shown in Fig 4B. In male mice, two genes participating in LPS biosynthesis, glycosyltransferase and UDP-perosamine 4-acetyltransferase, were up-regulated (Fig 4C). Moreover, Ace-K consumption increased the abundance of one bacterial toxin synthesis gene, thiol-activated cytolysin, as shown in Fig 4D. These data suggest that perturbation of the gut microbiome by Ace-K enriched the LPS synthesis-related genes, which might increase the risk of chronic inflammation in the host.

### 5. Ace-K significantly altered fecal metabolomes

**Fig 5:**
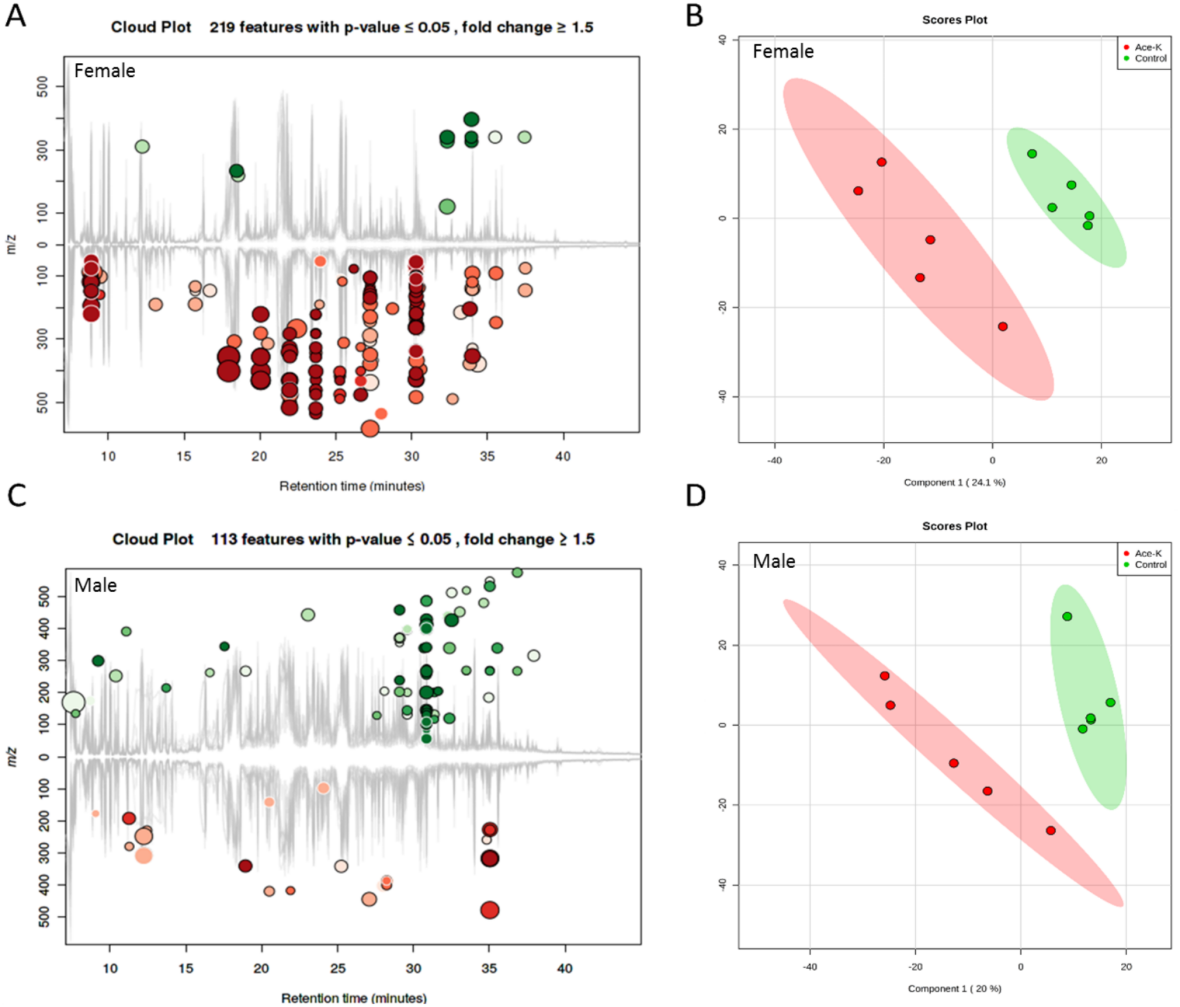
Ace-K consumption changed the fecal metabolome of female (A, B) and male (C, D) mice, as illustrated by the cloud and PLS-DA plots.

**Fig 6:**
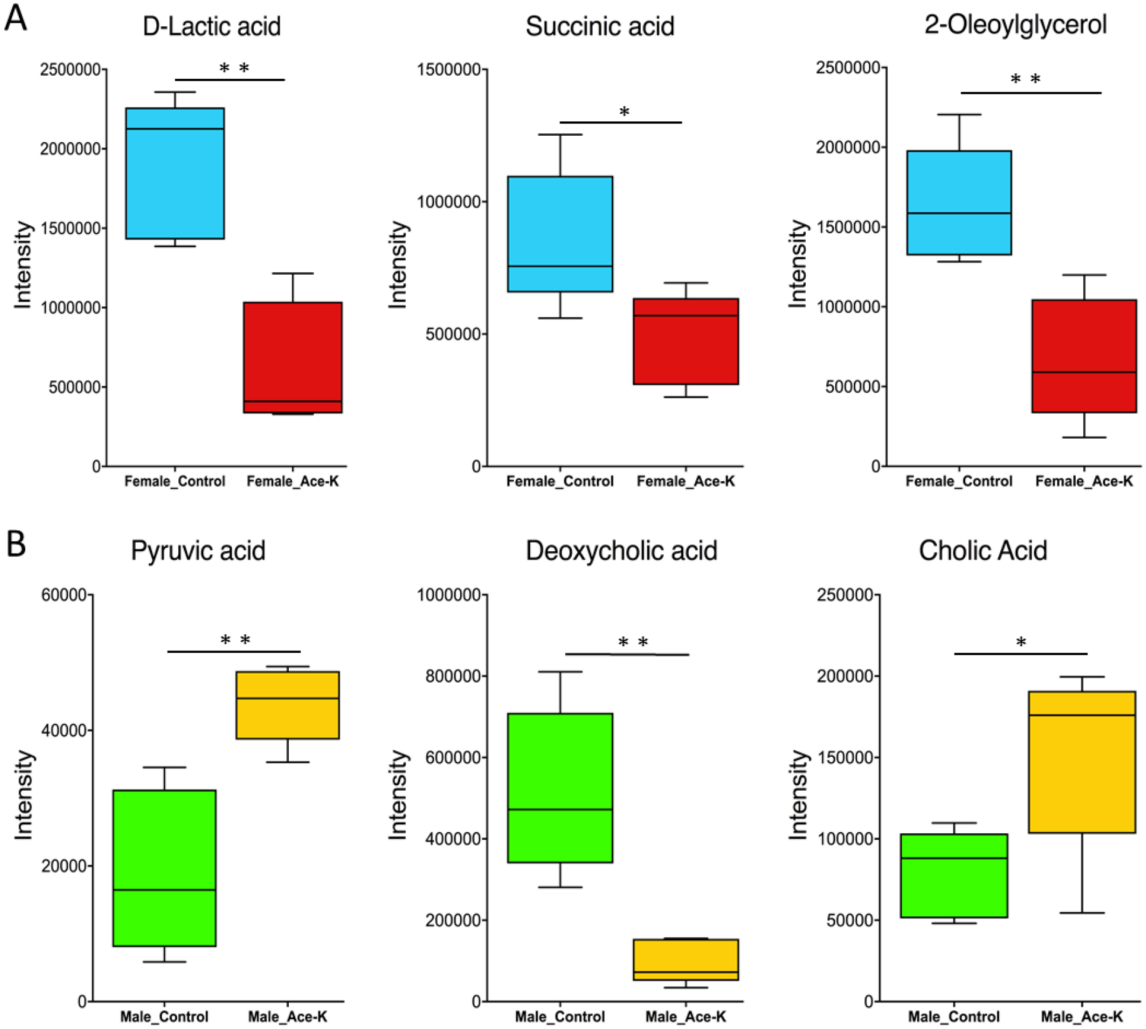
Ace-K consumption significantly altered key fecal metabolites in female (A) and male (B) mice (*p<0.05, **p<0.01).

Metabolites serve as signaling molecules for the complex crosstalk between the host and gut bacteria [7]. Therefore, we further investigated whether Ace-K consumption disturbed the fecal metabolic profiles. The cloud and PLS-DA plots shown in Fig 5 reveal that the gut microbial metabolomes of the Ace-K-administered animals were different from those of the controls, regardless of gender. However, the changes in specific metabolites were largely different in male and female mice, with the majority of metabolites being down-regulated and up-regulated in female and male mice, respectively, which was consistent with the differential changes in the gut bacterial community composition and functional genes in the female and male animals. S1 and S2 Tables list the identified metabolites that were significantly different between the control and treatment groups for female and male mice, respectively. Notably, multiple bacterial metabolism-related metabolites, such as lactic acid and succinic acid, were decreased in female mice, as shown in Fig 6A. 2-Oleoylglycerol (2-OG) was also largely decreased in females. In male animals treated with Ace-K, the concentration of pyruvic acid, a central metabolite of energy metabolism, was significantly higher than in the control animals (Fig 6B). Interestingly, cholic acid (CA) was increased, while deoxycholic acid (DCA) was dramatically decreased in the feces of male mice (Fig 6B). The fecal metabolomic profiling results suggested that Ace-K consumption can significantly change the gut metabolic profiles, which can influence the crosstalk between the host and gut microbiome.

## Discussion

In this study, we applied DNA sequencing and metabolomics approaches to characterize the gender-specific effects of Ace-K consumption on gut microbiota and their metabolism. Ace-K consumption altered the gut bacterial composition and metabolism profile, and the perturbations were highly gender dependent. Specifically, Ace-K increased the body weight gain in male but not female mice and induced different gut bacterial composition changes in male and female mice. In addition, functional gene enrichment analysis revealed a significant gender-specific effect, with numerous bacterial genes involved in energy metabolism being activated in male mice but inhibited in female animals. Moreover, Ace-K may also increase the risk of developing chronic inflammation by disrupting gut bacteria and associated functional pathways. This study provides novel insights into the effects of artificial sweetener consumption on host health and highlights the role of the gut microbiome and bacterial products in regulating host metabolic homeostasis. Previous studies focusing on the effects of artificial sweeteners, gut microbiota and host health have rarely considered the role of gender, but our results emphasize the importance of gender in mediating the gut microbiome and host response to compounds such as artificial sweeteners.

Although artificial sweeteners have been considered safe, accumulating evidence indicates that they can induce glucose intolerance and disturb energy homeostasis in the human body. In particular, the gut microbiome has been demonstrated to play a role in these processes [1, 3]. In this study, the components of the gut microbiome were altered in Ace-K-treated mice (Fig 1B and 1C). Notably, the abundance of the genus *Bacteroides* was much higher in male mice treated with Ace-K than in the controls. This result is consistent with a recent study on artificial sweeteners [3], which found that saccharin consumption could induce the over-growth of *Bacteroides* in male mice. *Bacteroides* is one of the most abundant and well-studied members of commensal microbiota. Many *Bacteroides* species have an extensive capability to utilize glycan and can produce fermentative end products, short-chain fatty acids (SCFAs), to supply nutrition and other beneficial properties to their host [9, 37]. Likewise, *Anaerostipes* also increased in abundance in Ace-K-treated male mice. As one of the major members of *Firmicutes*, the genus *Anaerostipes* contains multiple SCFA-producing species, such as *Anaerostipes butyraticus* sp. *nov*. and *Anaerostipes caccae* [38–40]. A high abundance of *Bacteroides* and *Anaerostipes* in the gut microbiome generally reflects a high capacity for energy harvesting and is associated with obesity [9]. Corresponding to the increase in these genera, the functional gene enrichment analysis indicated that a number of genes involved in carbohydrate absorption, degradation and fermentation were consistently increased in Ace-K-treated male mice. Moreover, pyruvate was also significantly increased in the male mice administered Ace-K (Fig 6B). Pyruvate is one of the key metabolites related to energy metabolism and can be further fermented to various SCFAs, such as propionate and butyrate [41]. Therefore, the significant increases in pyruvate, *Bacteroides* and *Anaerostipes*, as well as in the genes involved in energy metabolism pathways, suggest that the energy metabolism and harvesting capacity in male mice was increased by Ace-K consumption. These results are consistent with a significantly higher body weight gain in Ace-K-treated males compared to the controls (10.28 g vs 5.44 g, p<0.01).

Interestingly, the response of the gut microbiota in female mice was remarkably different or even opposite that in male mice. Four weeks of Ace-K consumption caused decreases in multiple genera of gut bacteria, including *Lactobacillus*, *Clostridium*, and unassigned genera in *Ruminococcaceae* and *Oxalobacteraceae*, as shown in Fig 1B. According to previous studies, bacteria in these genera play crucial roles in food digestion and polysaccharide fermentation [8, 42, 43]. For example, *Ruminococcaceae* is considered a primary member of the bacterial community that hydrolyzes complex food fibers for gut fermentation, which plays an important role in host energy utilization [44]. Likewise, some species of *Clostridium*, such as *Clostridium thermocellum*, can produce glycoside hydrolase and participate in polysaccharide digestion [8]. Moreover, functional gene enrichment analysis showed that carbohydrate absorption, degradation and fermentation pathways were significantly decreased in the female mice after Ace-K consumption (Fig 3). The significant decline of these bacterial components and functional genes indicated that Ace-K consumption impaired the polysaccharide digestion and fermentation ability of the gut microbiome in female mice, which could further influence energy harvesting in the host. This conclusion was further supported by the GC-MS data revealing that various fermentation products, such as lactic acid and succinic acid, were decreased in female mice treated with Ace-K (Fig 6A). Consequently, in contrast to the substantial increase in body weight gain in the male animals, Ace-K consumption did not significantly affect the body weight gain of female mice.

It has been well documented that toxic products generated by gut bacteria can enter systemic circulation and induce chronic inflammation [45–47]. Thus, disrupted gut microbiome could contribute to the development of chronic inflammation. In fact, our results support that the perturbation of gut bacteria induced by Ace-K may increase the risk of developing systemic chronic inflammation, which could be achieved through different ways, such as disrupting the gut bacterial composition, activating bacterial genes of pro-inflammatory mediators and perturbing functional metabolites. For example, *Bacteroides* and *Sutterella* were significantly increased in Ace-K-treated male mice (Fig 1C). A previous study found that multiple selected commensal species of *Bacteroides*, including *B*. *thetaiotaomicron* and *B*. *vulgatus*, could induce colitis in mice [48]. Similarly, a recent study found that *Sutterella* spp. have a pro-inflammatory capacity and an ability to adhere to intestinal epithelial cells, indicating a potential role in host immunomodulation [49]. Likewise, the functional gene enrichment analysis suggested that Ace-K-activated bacterial genes of pro-inflammatory mediators may increase inflammation. Toxic products or endotoxins, such as LPS, generated by gut bacteria can enter systemic circulation and induce chronic inflammation [45–47]. A significant increase in LPS synthesis and modification genes was observed after Ace-K consumption, especially in female mice (Fig 4A and 4C). Moreover, thiol-activated cytolysin, a prominent Gram-positive bacterial toxin and an important virulence factor [50, 51], was increased in Ace-K-treated male mice (Fig 4D). This toxin can stimulate the expression of inflammatory mediators, such as cytokines, and induce inflammatory responses [50, 51]. Finally, bile acids in fecal samples from Ace-K-treated male mice were affected (Fig 6B). Multiple previous studies have shown that normal bile acid metabolism plays a role in regulating the inflammatory response [52, 53]. In this study, we found that CA and DCA increased and decreased, respectively, after Ace-K treatment (Fig 6B), highlighting the effects of Ace-K on the homeostasis and biotransformation of bile acids, important signaling molecules in inflammation and glucose metabolism [54–57].

Collectively, our data evidence that Ace-K consumption can lead to adverse effects in the gut microbiome of mice. Notably, distinct gender-specific effects were observed in this study. Ace-K consumption led to a significant increase in body weight in male mice, potentially by disrupting the gut bacterial compositions and activating bacterial energy harvesting pathways. However, no significantly increased body weight gain was observed in female mice. Pathways related to energy metabolism were down-regulated in Ace-K-treated female mice, which could prevent the occurrence of the obese phenotype in female mice. Although the gut microbiome plays a key role in regulating host metabolism, we could not rule out other mechanisms that may also contribute to the Ace-K-induced alterations of energy homeostasis and body weight regulation in mice. For example, some studies have suggested that nonnutritive sweeteners could interfere with physiological responses that regulate energy homeostasis [2, 58]. Evidence also supports the idea that artificial sweeteners may interact with sweet taste-like receptors to influence incretin release and further disturb energy homeostasis [58, 59]. Taken together, the responses and complex regulations of the gut microbiome might be involved in energy metabolism of the host during artificial sweetener consumption. Future studies are warranted to further elucidate the mechanisms involved.

Several limitations are associated with this study. Although we observed a strong gender-dependent effect of Ace-K on the gut microbiome and body weight gain of mice, our results are based on a small sample size. Further validation in a larger number of animals or a human cohort is needed. Likewise, we did not measure food intake, body composition and relevant fat pads of mice, which represents another limitation of the study. In addition, we performed a 4-week exposure using a single high dose of Ace-K, while human exposure is frequently long term and at lower concentrations. Our ongoing study using multiple human-relevant doses aims at better understanding the chronic effect of Ace-K on the gut microbiome and host. Finally, the major goal of this study was to define the impact of Ace-K on the gut microbiome and fecal metabolome. Further characterization of the effect of Ace-K on the host physiology, such as the inflammatory response and energy homeostasis, would provide novel and important insights into Ace-K-induced metabolism perturbations in the gut microbiome and host.

## Supporting information

S1 Table Significantly altered metabolites (p < 0.05, compared to controls) identified in fecal samples from Ace-K-treated female mice. (PDF)

S2 Table Significantly altered metabolites (p < 0.05, compared to controls) identified in fecal samples from Ace-K-treated male mice. (PDF)
