# Supplementary material for: The artificial sweetener acesulfame potassium affects the gut microbiome and body weight gain in CD-1 mice
Source: PLoS One. 2017 Jun 8;12(6):e0178426. doi: 10.1371/journal.pone.0178426 (PMC5464538; doi:10.1371/journal.pone.0178426)
Supplement: S1 Table — (PDF) [file pone.0178426.s001.pdf]

S1 Table. Significantly altered metabolites ($p < 0.05$, compared to controls) identified in fecal samples from Ace-K-treated female mice.

Metabolites	Fold	p value	m/z	Retention time	Pathways or Functions
Phosphoric acid	2.43	0.034	310.1000	12.24	-
Urea	-1.35	0.035	132.1000	11.85	Urea cycle
Stearic acid	-1.40	0.018	271.2000	25.64	Fatty acids
Palmitic acid	-1.49	0.033	75.1000	23.40	Fatty acids
Isoferulic acid	-1.53	0.034	190.1000	23.92	Isoferulic acid
Linoleic acid	-1.58	0.014	475.0000	25.26	Fatty acids
Succinic acid	-1.74	0.031	133.1000	15.73	TCA cycle
Uracil	-2.28	0.039	190.1000	13.12	Pyrimidine
2-Oleoylglycerol	-2.44	0.0041	218.2000	30.28	Monoacylglycerol
Sitosterol	-2.50	0.024	247.2000	35.55	Sterol
Oleic acid	-2.58	0.041	340.3000	30.31	Fatty acids
Lactic acid	-2.87	0.017	240.3000	29.08	Fermentation
Cholesterol	-2.96	0.031	143.1000	33.99	Sterol
2-Pentanone	-3.12	0.0017	76.1000	8.87	-
N-Acetyl-D-glucosamine	-4.14	0.027	282.1000	23.67	Glucose derivative
Tyrosine	-4.75	0.0049	356.1000	20.04	Amino acids
