# Supplementary material for: The artificial sweetener acesulfame potassium affects the gut microbiome and body weight gain in CD-1 mice
Source: PLoS One. 2017 Jun 8;12(6):e0178426. doi: 10.1371/journal.pone.0178426 (PMC5464538; doi:10.1371/journal.pone.0178426)
Supplement: S2 Table — (PDF) [file pone.0178426.s002.pdf]

**S2 Table.** Significantly altered metabolites ($p < 0.05$, compared to controls) identified in fecal samples from Ace-K-treated male mice.

| Metabolites | Fold | p value | m/z | Retention time | Pathways or Functions |
|---|---|---|---|---|---|
| Pyruvic acid | 2.31 | 0.0065 | 300.1000 | 9.22 | Fermentation |
| Cholic acid | 1.92 | 0.044 | 185.2000 | 34.96 | Bile acids |
| α-Glyceryl stearate | 1.87 | 0.0064 | 83.2000 | 30.85 | Glycerolipids |
| 1,3-Dipalmitin | 1.85 | 0.017 | 240.3000 | 29.08 | Glycerolipids |
| Glutamic acid | 1.73 | 0.0097 | 345.2000 | 17.54 | Amino acids |
| Linoleic acid | 1.33 | 0.048 | 263.3000 | 25.28 | Fatty acids |
| Phosphoric acid | -1.32 | 0.047 | 119.1000 | 12.13 | - |
| Cholesterol | -1.47 | 0.034 | 142.2000 | 34.01 | Sterol |
| Lysine | -1.60 | 0.021 | 419.3000 | 21.89 | Amino acids |
| Glycolic acid | -1.61 | 0.039 | 178.1000 | 9.05 | - |
| Campesterol | -1.73 | 0.044 | 260.3000 | 34.82 | Sterol |
| Isoleucine | -1.90 | 0.035 | 231.2000 | 12.42 | Amino acids |
| Ornithine | -2.23 | 0.036 | 142.2000 | 20.49 | Amino acids |
| Deoxycholic acid | -5.33 | 0.0084 | 319.2000 | 35.04 | Bile acids |
